# Development of Light, Strong, and Water-Resistant PVA Composite Aerogels

**DOI:** 10.3390/nano14090745

**Published:** 2024-04-24

**Authors:** Amir Abdolazizi, Ishara Wijesinghe, Ifra Marriam, Hiran Chathuranga, Dmitri Golberg, Cheng Yan

**Affiliations:** 1 School of Mechanical, Medical and Process Engineering, Queensland University of Technology (QUT), Brisbane, QLD 4000, Australia; amir.abdolazizi@hdr.qut.edu.au (A.A.); ishara.gedara@hdr.qut.edu.au (I.W.);; 2 Centre for Materials Science, Queensland University of Technology, Brisbane, QLD 4000, Australia; 3 Department of Chemical and Process Engineering, Faculty of Engineering, University of Moratuwa, Moratuwa 10400, Sri Lanka; 4 School of Chemistry and Physics, Faculty of Science, Queensland University of Technology (QUT), Brisbane, QLD 4000, Australia

**Keywords:** anisotropic aerogel insulators, solvent-assisted freeze-casting, ultralow density, compressional modulus

## Abstract

A significant weakness of many organic and inorganic aerogels is their poor mechanical behaviour, representing a great impediment to their application. For example, polymer aerogels generally have higher ductility than silica aerogels, but their elastic modulus is considered too low. Herein, we developed extremely low loading (<1 wt%) 2D graphene oxide (GO) nanosheets modified poly (vinyl alcohol) (PVA) aerogels via a facile and environmentally friendly method. The aerogel shows a 9-fold increase in compressional modulus compared to a pure polymer aerogel. With a low density of 0.04 mg/mm^3^ and a thermal conductivity of only 0.035 W/m·K, it outperforms many commercial insulators and foams. As compared to a pure PVA polymer aerogel, a 170% increase in storage modulus is obtained by adding only 0.6 wt% GO nanosheets. The nanocomposite aerogel demonstrates strong fire resistance, with a 50% increase in burning time and little smoke discharge. After surface modification with 1H,1H,2H,2H-Perfluorodecyltriethoxysilane, the aerogel demonstrates water resistance, which is suitable for outdoor applications in which it would be exposed to precipitation. Our research demonstrates a new pathway for considerable improvement in the performance and application of polymer aerogels.

## 1. Introduction

Aerogels, characterized by their ultra-lightweight and solid form, are composed of three-dimensional porous networks. Their unique properties include high porosity, a large specific area, and very low acoustic and heat transfer. These make them suitable for various applications, including Electromagnetic Interference (EMI) shielding [1], energy storage [2], environmental protection [3], and high-temperature thermal insulation [4]. Up to now, a wide range of aerogels has been developed using different materials, including ceramics [5,6], metal/semiconductor [7], metal/ceramic composites [8], super-molecules [9], carbon/nanocarbon [10], and boron nitride [11]. However, the brittleness and low elasticity of aerogels could lead to structural collapse, which limits their practical applications [12]. Silica aerogel, despite being a highly promising insulation material, is hindered by its fragile mechanical properties stemming from its ceramic composition, leading to an easy breakdown and the release of hazardous dust [13,14,15]. In contrast, polymer aerogels have higher ductility, which makes them suitable candidates for practical applications such as thermal insulators [16,17], packaging and furniture cushioning materials [18], and pressure-temperature sensors [19], but they suffer from low strength and compressive modulus [20,21].

Conventional thermal insulating materials, being isotropic, are less effective in heat conduction mitigation. Therefore, they should be used in greater thicknesses to achieve satisfactory insulation efficiency [22,23]. Furthermore, research and development in this area are stagnating and any further improvement in thermal insulation would come at the expense of mechanical strength, ease of manufacturing, and performance stability [24]. Consequently, there is a growing focus on the development of anisotropic thermal insulators. For example, recently, “nanowood” was introduced using the direct chemical treatment of natural wood, resulting in a thermally insulating bulk material with a conductivity of 0.06 W/m·K along the cellulose alignment [25]. Nevertheless, this bottom-up method faces limitations in its adaptability to different materials. 

Freeze casting is an environmentally friendly approach to create porous structures with the desired alignment and versatility in the obtained structure [26,27]. While being a straightforward and cost-effective method for polymer aerogel production, the resulting aerogels are fragile due to their thin cell walls [28,29]. Consequently, polymer nanocomposite aerogels, offering enhanced shape stability, have been introduced. However, typically high nanofiller loadings are used. For example, Chan et al. produced decimetre-scale polyurethane aerogel panels that had a strength of only 7.37 kPa along the axis, although they incorporated 10 wt% boron nitride Nanosheets (BNNS) [23]. Wicklein et al. engineered anisotropic foams using nanocellulose and graphene oxide (GO) via freeze casting, achieving a Young’s modulus of 570 kPa at 23 wt% of nanofillers [4]. The poly(vinyl alcohol) (PVA)/cellulose nanofibers aerogel developed by Huang et al. through freeze drying showed a Young’s modulus of less than 0.4 MPa, although incorporating 60 wt% of nanofillers [30].

In this study, we innovatively develop anisotropic aerogel structures with customized mechanical properties, utilizing a minimal quantity of GO nanosheets compared to conventional polymer nanocomposites. This approach significantly enhances both the mechanical and thermal characteristics, while maintaining the aerogel’s low-density structure. Remarkably, the aerogel achieves a competitive low heat conductivity of 0.035 W/m·K. Mechanical and thermomechanical tests reveal notable improvements in the strength and storage modulus, with a compressional modulus of 550 kPa and an enhanced thermal stability up to 120 °C. Post-synthesis surface modifications render the aerogel water-resistant, which is suitable for applications where the aerogel is exposed to precipitation. This research represents a pioneering effort in achieving mechanical and thermal performance enhancements in highly anisotropic polymer nanocomposite aerogels using extremely low levels of additives. 

## 2. Experimental Methods

### 2.1. Materials and Methods

**Figure 1:**
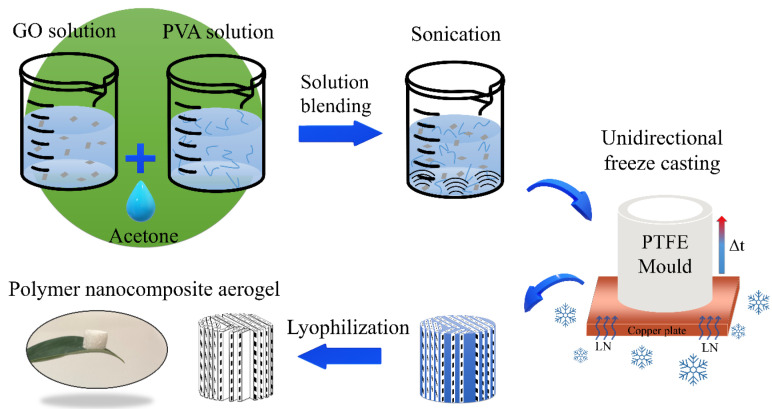
The schematic representation of nanocomposite polymer aerogels synthesis.

The synthesis of PVA nanocomposite aerogels is schematically shown in Figure 1. GO was synthesised through a modified Hummers method [31]. GO nanosheets were obtained from bulk GO through liquid phase exfoliation in an ultrasonic ice bath (Branson b2500R-MTH, 85 W, 42 kHz) for 1 h until a uniform dispersion was achieved. A PVA (Sigma-Aldrich, MW 146,000–186,000, 99+% hydrolysed) solution (2 wt%) was obtained by dissolving the powder in hot water for 1 h using a magnetic stirrer. The dispersion of GO nanosheets in PVA was carried out through a solution blending method. Different combinations of the solutions (Table 1) were mixed and stirred using a magnetic stirrer for 3 h followed by sonication for 1 h to obtain a homogenous dispersion. Unidirectional freeze casting was conducted using a piece of custom-designed equipment. The dispersion was poured into a polytetrafluoroethylene (PTFE) mould with a copper plate bottom. The mould was then put onto a copper cold source which was in contact with liquid nitrogen. The liquid nitrogen created a large temperature gradient and induced the rapid unidirectional growth of ice crystals. The samples were transferred to a freeze dryer with a chamber pressure of 8 Pa and a temperature of −44 °C. Aerogels were obtained after lyophilisation for at least 48 h. Acetone-assisted synthesis was carried out through the same method with the addition and mixing of 4 vol% of acetone to the dispersion before freeze casting. Chemical surface modification was carried out by dispersing 0.5 g 1H,1H,2H,2H-Perfluorodecyltriethoxysilane (Sigma-Aldrich, Mountain Highway, Bayswater, Australia, 97%) in 10 mL ethanol and immersing the aerogel in the solution for 10 min, followed by drying at 135 °C for 30 min. The water-resistant PVA nanocomposite aerogel was obtained.

### 2.2. Characterization

The porosity of the aerogels was determined using Equation (1):

(1) $$\mathrm{Porosity}=\left(1-\frac{\rho }{\rho_{0}}\right)\times 100\;\%$$

where *ρ* is the apparent density of the aerogel and *ρ*_0_ is the density of an equivalent solid nanocomposite determined from the weighted average densities of GO (1.91 g/cm^3^) and PVA (1.19 g/cm^3^). Atomic force microscopy (AFM, Bruker Dimension Icon, Bruker, Billerica, MA, USA) was carried out to measure the thickness of GO nanosheets. The samples were deposited on a clean silicon substrate from an exfoliated GO solution. Scanning electron microscopy (SEM, TESCAN MIRA3, TESCAN, Brno, Czech Republic) was conducted at an operating voltage of 8 kV to characterise the morphology of the aerogels. Fourier transform infrared spectroscopy (FTIR, 5700 Nicolet Diamond AT, Bruker Dimension Icon, Bruker, Billerica, MA, USA) was conducted to examine the chemical composition of the aerogels over wavenumbers of 500–4000 cm^−1^. The crystal structure of the aerogels was analysed through X-ray diffraction spectroscopy (XRD, Bruker D8). X-ray photoelectron spectroscopy (XPS, Kratos AXIS Supra, Kratos, Manchester, UK) was conducted to examine the elemental composition and chemical structure of the aerogels. The CasaXPS v2.3.25PR1.0 software program was used for curve fittings. The thermal insulation of the aerogels along the axial direction was examined by placing the aerogels on a ceramic hot plate (60 °C–120 °C), and the changes in temperature at the top of the samples were monitored using an infrared camera (FLIR i7, Teledyne FLIR, Mulgrave, Australia). The thermal conductivity of the aerogels along the axis was determined using laser flash analysis (LFA, Netzsch LFA 467 Laser Hyperflash, Netzsch, Selb, Germany) at room temperature. This instrument can measure the thermal diffusivity (*α*) and specific heat (*Cp*) of the samples at a given temperature. The thermal conductivity (*λ*) could then be determined using Equation (2) [32]:

(2) $$\lambda \left(T\right)=\alpha \left(T\right)\cdot C_{P}\cdot \rho (T)$$

The flame response of the aerogels was monitored by exposing the aerogels to a lit candle. Air ventilation was minimised in the testing chamber to increase smoke visibility. The response of the aerogels to water was assessed by dropping deionised water droplets on the top surface of the aerogels. Thermogravimetric analysis (TGA, NETZSCH STA 449F3) was performed to understand the thermal stability of the aerogels. The samples were examined at a temperature ramp of 10 °C/min from room temperature to 800 °C in alumina crucibles with lids. All mechanical tests were performed using a rheometer (Anton Paar MCR302, Anton Paar, Graz, Austria) equipped with a pair of parallel compression plates. For all compression tests, the diameter of the plate was 25 mm, and the movement speed of the upper plate was equal to 1 m/s, under nominal room conditions. For oscillatory temperature ramp experiments, the diameter of the plate was 10 mm, and the normal applied force was 2 N. The strain was within the linear viscoelastic region (=0.1%), and angular frequency was set at 1 rad/s. The sample temperature was increased from room temperature to 180 °C at a heating rate of 10 °C/min.

## 3. Results and Discussion

### 3.1. Characterisation of GO Nanosheets and Nanocomposite Aerogels

The GO nanosheets were exfoliated through sonication in an ice bath, resulting in a uniformly dispersed solution in deionised water. SEM and AFM observations confirm that the nanosheets consist of a few atomic layers (Figure S1). The thickness of the GO nanosheets was estimated to be around 4 nm from AFM micrographs. Considering the thickness of a monolayer GO nanosheet (≈1 nm) [25], the successful exfoliation from the bulk was confirmed. 

**Figure 2:**
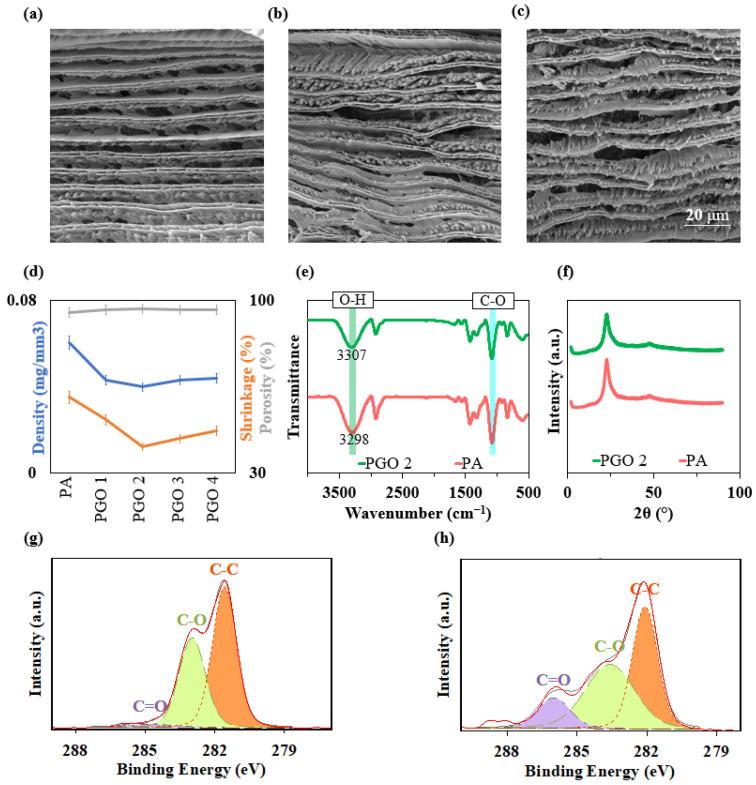
SEM microstructure of (**a**) PA, (**b**) PGO 1, and (**c**) PGO 2 aerogels. (**d**) Density, shrinkage, and porosity of aerogels. (**e**) FTIR spectra and (**f**) XRD patterns of PA and PGO 2. C1 S XPS spectra of (**g**) PA and (**h**) PGO 2.

The morphology of the aerogels was investigated by SEM. Figure 2a–c show highly anisotropic channels where cells are aligned along the axis of the samples. The cell walls were formed by the expelling of the solid particles in the solution during freezing, which were confined between the growing ice fronts. The elongated pores were formed by the sublimation of the ice crystals during vacuum drying. 

Figure 2d compares the density, shrinkage, and porosity of the PVA-GO nanocomposite aerogels. All the aerogels exhibit low densities due to a very low solid content. Interestingly, the nanocomposite aerogels have lower densities than PA. Most notably, the density of PGO 2 (0.04 mg/mm^3^) is 33% lower than the density of PA (0.06 mg/mm^3^). Observably, the addition of 2D nanofillers reduced the density of the polymer aerogel, although the density of the GO nanosheets is higher than that of the PVA matrix. This could be due to the improved shape stability and suppressed shrinkage by the addition of strong GO nanosheets. The rigid and stiff GO nanosheets reinforced the polymer walls and limited the shrinkage for all nanocomposite aerogels, with a minimum shrinkage of 40.6% achieved with 0.6 wt% GO loading (i.e., PGO 2). By maintaining most of the original dimensions, the nanocomposite aerogels show reduced densities. Similarly, the porosity of PA (=94.9%) increased to 96.65% by the addition of 0.6 wt% GO nanosheets. The GO nanosheets were less effective in decreasing the density of the aerogels when added at the amounts of 0.9 wt% (PGO 3) and 1.2 wt% (PGO 4), where the densities of aerogels were equal to 0.043 mg/mm^3^ and 0.044 mg/mm^3^, respectively. This is likely to be an effect of the addition of large amounts of heavy GO nanoparticles and the tendency of nanosheets to agglomerate [33]. Therefore, PGO 2 was chosen in this study as the nanocomposite with the optimum GO loading. 

The integration of the GO nanosheets in the PVA matrix and their bonding were in-vestigated using FTIR, XRD, and XPS. Figure 2e compares the FTIR spectra of the PA and PGO 2 aerogels. Both samples share the characteristic peak of PVA at 1077 cm^−1^, which indicates C–O stretching [34,35]. However, the broad band between 3000–3700 cm^−1^, which is attributed to the stretching vibration of –OH is shifted to higher wave-numbers by the addition of GO nanosheets. Also, the C–O absorption peak shows a stronger intensity in PA compared to PGO 2, indicating effective hydrogen bonding be-tween the hydroxyl groups of PVA and the oxygen-containing functional groups of GO nanosheets [36,37]. This bonding effectively increases the interfacial interactions between the GO nanofillers and the PVA matrix. The XRD spectra of PA show a characteristic peak at 23°, which corresponds to the (101) crystalline plane of the polymer (Figure 2f). The addition of GO nanosheets does not change this pattern, indicating the effective dis-persion of nanoparticles within the matrix [38,39]. XPS spectra also demonstrate effective bonding between the PVA matrix and GO nanofillers. The deconvolution of the C1s peak of PA in Figure 2g shows 3 distinct peaks that can be assigned to C-C, C-O, and C=O. C-C and C=O bonds that comprise the structure of GO nanosheets show stronger peaks in the spectra of PGO 2, indicating the successful incorporation of GO nanosheets into the PVA matrix (Figure 2h).

### 3.2. Thermal Properties and Environmental Stability of the Nanocomposite Aerogel

**Figure 3:**
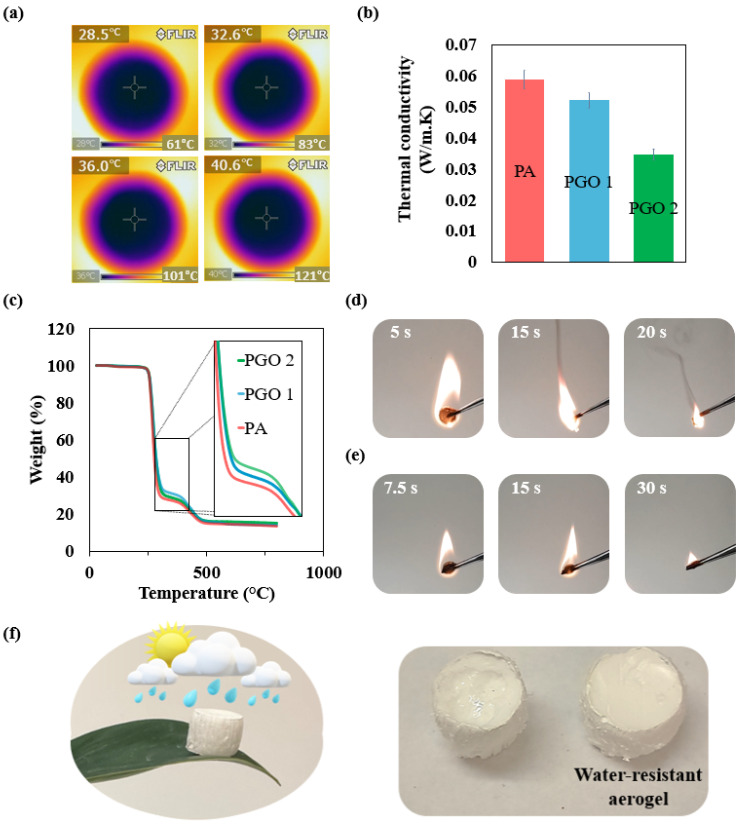
(**a**) IR thermal images of PGO 2 on a hot plate. (**b**) Thermal conductivity of aerogels. (**c**) TGA curves of aerogels. Flame response of (**d**) PA and (**e**) PGO 2. (**f**) The water resistance of PGO 2.

Nowadays, a wide range of applications requires high-performance thermal insulation materials in space, building, and energy industries [40]. Thermal insulation performance tests were carried out by placing PGO 2 on a hot plate and monitoring the temperature evolutions. IR thermal images are shown in Figure 3a. Considerable differences in the temperatures of the top of the aerogel and the hot plate were observed. The measurements were carried out 4 times at hot plate temperatures ranging between 60 °C and 120 °C, and there was an average of 61% difference in the temperatures of the top surface of the aerogel and the hot plate surface, which reflects the high resistance of PGO 2 to heat transfer.

The thermal conductivities of the samples were tested using laser flash analysis (Figure 3b). All the aerogels show thermal conductivities between 0.035–0.059 W/m·K which are in the range of commercially available insulators (see Table 2). Moreover, the test results show diminishing thermal conductivity with the addition of GO nanofillers. The conductivities of PGO 1 and PGO 2 were 12% and 41% less than PA, respectively. The results indicate that the thermal conductivity of PGO 2 is less than a range of commercial insulators and foams. Also, a comparison with similar PVA composite insulators reveals that PGO 2 outperforms many of them, despite using a small amount of nanofiller loading.

The reason behind this great thermal performance should be attributed to the combined effects of the anisotropic porous structure and the ultralow solid content of the presented aerogels. Thermal conductivity in foams is the result of the total heat transfer through air convection (*λ_conv_*), radiation (*λ_rad_*), solid conduction (*λ_s.cond_*), and gas conduction (*λ_g_*_.*cond*_), according to Equation (3) [4]:

(3) $$\lambda =\lambda_{conv}+\lambda_{rad}+\lambda_{g.cond}+\lambda_{s.cond}$$

The contribution of air convection is negligible due to the scale of pore sizes, which are much smaller than the critical 1 mm required for natural convection to occur [49]. The effect of radiation is also marginal at the test temperature and the low density of the aerogels [50]. Heat conduction through gas is impeded by the Knudsen effect, which occurs when the movement of gas molecules is constrained within pores that are smaller than their mean free path [51]. Air conduction and convection were further limited by the isolated nature of the pores in the channelled structures [52].

It could then be concluded that the thermal conductivity in the current aerogels is dominated by the quality of heat conduction in the solid pore walls. The structure of PGO 2 was engineered to lower solid conductivity for a number of reasons: firstly, the aerogel’s extremely low solid content, consisting of just 20 mg/mL PVA which contained 0.6 wt% GO nanosheets, significantly reduced the total heat transfer in the solid due to fewer conduction pathways. Additionally, incorporating GO nanosheets in PGO 2 resulted in a 33% reduction in density, which further diminished solid conduction [5,53]. Furthermore, the addition of GO nanosized components induced a significant interfacial thermal resistance known as Kapitza resistance, which reduced the thermal conduction in the cell walls [54,55]. Lastly, the reduction of bridges between cell channels induced more phonon scattering, further reducing solid thermal conduction [20], and endowing our aerogel with significant thermal resistance close to that of air (≈0.03 W/m·K) [56]. 

The thermal stability of the nanocomposite aerogels was evaluated by TGA. The results in Figure 3c show that PA experienced a two-stage degradation, comprising a drastic weight loss between 250–325 °C, resulting from the melting of the polymer, and a minor weight loss between 375–500 °C, resulting from the decomposition of polymer chains [57]. The PGO 1 and PGO 2 curves do not show any appreciable differences from PA. This demonstrates the significance of the structure of the nanocomposite aerogels when it comes to the thermal properties, rather than their mere chemical composition, which was very close to that of a pure polymer aerogel due to extremely small 2D filler loadings. Even so, the slopes of PGO 1 and PGO 2 curves are slightly lower, between 250–325 °C, indicating the mass loss in the nanocomposites took place more slowly than for the pure polymer aerogel. This suggests that the GO nanosheets acted as thermal barriers even at an extraordinarily low amount [58].

Flame tests were carried out on PA and PGO 2 to assess their response to fire. As depicted in Figure 3e, PGO 2 shows a long and stable burning compared to PA (Figure 3d, Supplementary Videos S1 and S2). While PA burns fiercely with a bright glow, PGO 2 hardly casts any glow. Also, unlike PA, PGO 2 emits very little smoke, which could reduce the adverse health effects of smoke in case of fire [59]. This could be attributed to the physical barrier effect of GO nanosheets. These nanosheets form a char layer that serves as a thermally insulating barrier, reducing gas and heat diffusion during combus-tion. Therefore, it is demonstrated that GO nanosheets with a high aspect ratio and good thermal stability can help to prevent material consumption at a very low level of addi-tion [60,61].

The water resistivity of PGO 2 before and after surface modification was assessed. The test was carried out by dropping water droplets on the top surface of the aerogel and monitoring the interaction (Figure 3f). Before surface modification, the aerogel absorbed water droplets that were dropped on the top surface within 5 s. More importantly, polymer molecule chains were dissolved in water, which left spherical impressions in the shape and locations of the droplets on the top surface. In contrast, after surface modification, the aerogel proved impervious to water, and water droplets only accumulated on the top surface without being absorbed (Supplementary Video S3). PVA is water soluble and contains a large number of hydroxyl groups which make it a hydrophilic material. It could be concluded that due to the low surface energy of the 1H,1H,2H,2H-Perfluorodecyltriethoxysilane coating the affinity between PVA nanocomposite aerogel and water was reduced [33]. The water pool on the top surface moved by tilting the aerogel in different directions and could be easily poured off. In outdoor applications, this could wash off any potential dust or pollutants, indicating the ease in cleaning. The remaining moisture could be conveniently dabbed off using a commercial tissue or evaporate over time. At the end of the test, no changes on the top surface could be observed.

### 3.3. Mechanical Properties of the Nanocomposite Aerogels

**Figure 4:**
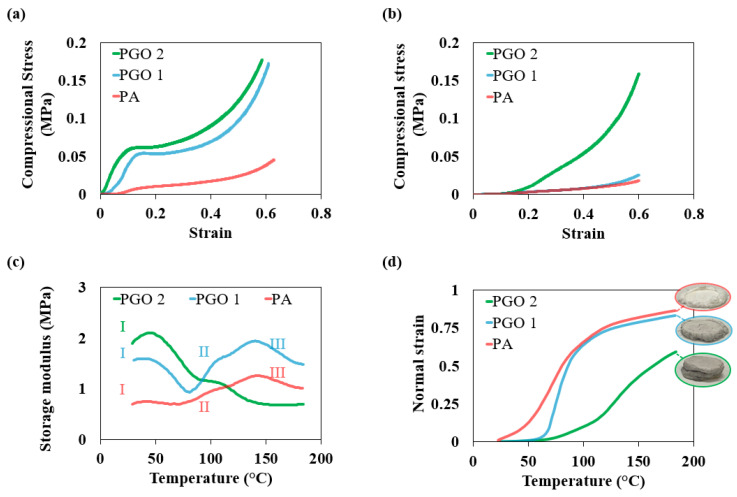
Compressive stress-strain curves of aerogels along (**a**) axial and (**b**) transverse directions. Evolutions of (**c**) storage modulus and (**d**) normal strain of aerogels during thermomechanical tests.

Sufficient mechanical properties are an important criterion for the selection of thermally insulating materials in any application for convenient storage and transportation [23]. The mechanical performance of the aerogels was assessed through uniaxial compression. The tests were carried out in both the axial and transverse directions of the aerogels up to a strain of 60%. The results along the axial direction of the aerogels are shown in Figure 4a. The compressive stress-strain curves of all the aerogels can be divided into 3 distinct regions: first, the linear elastic region, where the strength of the samples is linearly proportional to the applied force, followed by a plateau caused by the buckling of the thin cell walls, which were aligned parallel to the direction of the force. As a result of buckling, large amounts of deformation (≈16%) took place without a considerable increase in stress. Finally, the densification and flattening of aerogels occurred, where the stress dramatically increased with strain. The curves clearly show the increase of compressive modulus with the addition of ultralow amounts of GO nanosheets. The compressive modulus of PA increased from only 55 kPa to 385 kPa and 550 kPa with the addition of 0.3 wt% and 0.6 wt% GO nanosheets, respectively. This is because of the effective load transfer between the polymer matrix and the rigid nanofillers due to effective bonding and the improved bending stiffness of the cell walls with the addition of GO nanosheets. Although the reduction in density is typically accompanied by reduced strength, the strength of PGO 2 shows a 9-fold increase compared to PA, which renders it stronger than a range of commercial closed cell foams (122 kPa compared to 55–124 kPa at 50% strain) [52].

This remarkable increase in stiffness is due to the high alignment of GO nanosheets with the cell walls that were arranged during the freezing [23]. Due to the anisotropy of 2D nanosheets, they show higher strength when loaded along the in-plane directions, as opposed to having random orientations, which are usually obtained by other methods [62,63]. This plays a key role in establishing a stiff structure. Moreover, the flexibility of the PVA matrix is maintained due to the ultra-low filler concentration and the uniform distribution of the GO nanosheets (Figure S2) [64]. This is manifested by the samples’ densification, flattening, and flow at large strains. Conversely, upon loading along the transverse direction, where nanosheets were not oriented along the compressive force, the load-bearing effect of the nanofillers was less significant (Figure 4b). The compressive moduli of PGO 1 and PGO 2 show a modest improvement of 32% and 33%, respectively, compared to PA. This demonstrates the significance of the orientation of nanofillers in the design of nanocomposites. 

Thermomechanical tests were carried out to assess the mechanical robustness of the aerogels at higher temperatures. The aerogels were tested under combined shear and compressive loads under a temperature ramp, and the results are demonstrated in Figure 4c,d. At room temperature, the storage moduli of PGO 1 and PGO 2 were 116% and 175% higher than PA respectively, which resulted from the strengthening effect of GO nanosheets and the strong interaction between the PVA matrix and GO nanoparticles, in agreement with the results of compression tests. These high storage moduli suggest that the GO nanosheets effectively enhanced the crosslinking of PVA molecule chains [65]. During the temperature ramp, the curves of PA and PGO 1 show a similar 3-stage pattern: I. collapse and/or buckling, II. densification, and III. flattening. During the first stage, the storage moduli of both aerogels dropped since the robustness of the aerogels declined with increasing temperature. PA and PGO 1 showed an increase in the modulus after 64 °C and 72 °C, respectively, which is correlated with mechanical compression and densification. From Figure 4d it could be observed that the corresponding normal strain of these aerogels at these temperatures were 28% and 20%, respectively, indicating significant densification. Another drop in these curves is observed after 135 °C and 132 °C, respectively, which is related to the flattening and the flow of the sample out of under the punch. For PGO 2, however, the normal strain did not exceed 20% until 120 °C. This aerogel showed a steady and extended collapse with no increase in the storage modulus over this temperature range. Such a decreased rate of degradation with temperature was previously reported for fibrous PVA-carbon nanotube (CNT) nanocomposites but with a much higher content of nanofillers (20 wt%) [66]. Also, the normal strain of PGO 2 rose to only 58% by the end of the test, and no flow out of under the punch could be observed, which indicates that compression took place at a slower pace. This observation proves the effectiveness of a very small amount of GO nanosheets in enhancing the structural stability of PVA aerogel at elevated temperatures. Our results demonstrate that with increasing thermal resistance, nanocomposites with ultralow nanosheet fillers can maintain their mechanical stability over a broader temperature range.

### 3.4. Effects of Acetone on Morphology and Mechanical Properties of Aerogels 

**Figure 5:**
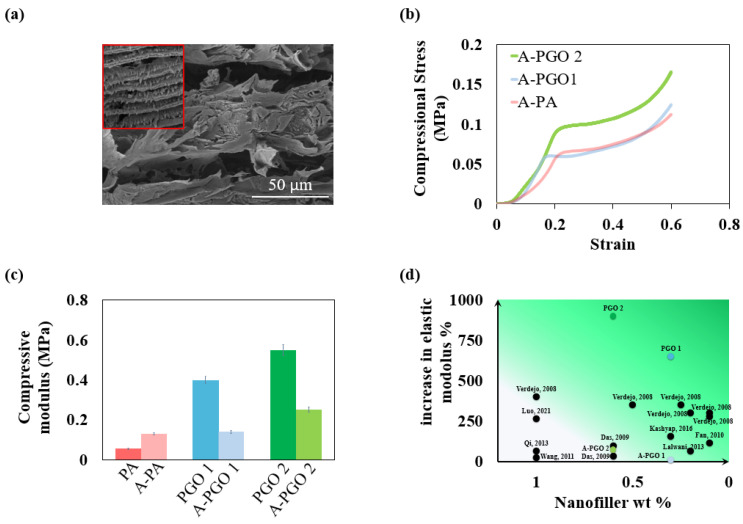
(**a**) SEM image of A-PGO 2 along the axial direction, the inset shows the SEM image of PGO 2 at the same magnification. (**b**) Compressive stress-strain curves of aerogels along the axial direction with the addition of acetone. (**c**) Comparison of axial compressive modulus of aerogels synthesized with and without acetone. (**d**) Comparison of axial compressive modulus of aerogels with the literature [68,69,70,71,72,73,74,75,76].

To investigate the effects of morphology on the properties of the GO-PVA nanocomposite aerogels, an acetone-assisted synthesis was carried out. The addition of acetone to the precursor before freezing resulted in significant microstructural changes. By functioning as an anti-freezing agent, the acetone limited the progression of the ice crystals [22]. This gave rise to smaller frozen water areas during freezing, and the portion of areas containing PVA polymer chains and GO nanosheets increased. After lyophilisation and the removal of the ice crystals, the large areas containing polymer molecules and GO nanosheets formed thick cell walls, and larger lamella spacings were observed. This was evidenced by SEM images (see Figure 5a and Figure S3). 

Compression tests were carried out on the aerogels with acetone-assisted freeze-cast synthesis (Figure 5b), and the compression moduli of aerogels with and without acetone are compared in Figure 5c. The modulus of PA shows a 136% improvement with the addition of acetone, which should be attributed to the increased bending rigidity of the thick cell walls. However, the increase of the modulus with the addition of the GO nanosheets was less tangible when an acetone-assisted approach was adopted. A-PGO 1 and A-PGO 2 show only 7.7% and 92% improvement in compressional modulus, respectively. It could be concluded that the thin cell walls of PGO 1 and PGO 2 result in a more compact stacking of GO nanosheets with PVA molecule chains, and therefore result in higher mechanical properties [67]. Comparison with the literature demonstrates that the compressive moduli of PGO 1 and PGO 2 are one of the highest recorded among polymer nanocomposites with similar loadings of graphene or GO.

## 4. Conclusions

In this work, we successfully developed lightweight and robust PVA nanocomposite aerogels through a facile and environmentally friendly method. A highly anisotropic structure with a low density of 0.04 mg/mm^3^ is achieved by the addition of only 0.6 wt% GO nanosheets to the PVA matrix (PGO 2), due to the improved shape stability and reduced shrinkage. A very low thermal conductivity of 0.035 W/m·K close to that of air (≈0.03 W/m·K) is achieved. This is because of limited conduction pathways and the reduced solid thermal conductivity of the polymer aerogel by the addition of GO nanosheets. The environmental stability of the nanocomposite is enhanced after chemical surface modification with 1H,1H,2H,2H-Perfluorodecyltriethoxysilane. Consequently, the PGO 2 nanocomposite aerogel demonstrates excellent water resistance and ease in cleaning. It also exhibits enhanced flame resistance, where a stable burning (1.5 times longer) is observed compared to pure PVA aerogel (PA), and hazardous smokes are reduced due to the barrier effect of GO nanosheets. A compressive modulus of 550 kPa (10 times higher than PA) is obtained as a result of improved bending stiffness of the thin cell walls. Thermomechanical tests demonstrate the increased thermal resistance of PGO 2, which resists the deformation up to 120 °C. Hence, this work may open a new way to manufacture high-performance aerogels. 

## Figures and Tables

**Table 1:** Abbreviation and composition of the aerogels.

Sample Name	Poly (Vinyl Alcohol) (PVA) (wt%)	Graphene Oxide (GO) (wt%)	Acetone (vol%)
PA	100	0	0
PGO 1	99.7	0.3	0
PGO 2	99.4	0.6	0
PGO 3	99.1	0.9	0
PGO 4	98.8	1.2	0
A-PA	100	0	4
A-PGO 1	99.7	0.3	4
A-PGO 2	99.4	0.6	4

**Table 2:** Comparison of the thermal conductivity of PGO 2 with different insulators, foams, and PVA aerogels.

	Insulator	Thermal Conductivity (W/m·K)	Reference
	PGO 2	0.035	Current study
Commercial insulators	Hemp	0.042	[41]
	Hempcrete	0.179	[42]
	Glass mineral wool	0.03–0.04	[22]
	Fiberglass	0.033–0.044	[22]
	Cork	0.04–0.05	[22]
	Cellulose insulation	0.04–0.05	[43]
Foams	XPS	0.037	[44]
	EPS	0.036	[44]
	PUR	0.031	[44]
	PE	0.039	[44]
	Coal fly ash composite foam	0.051	[45]
PVA aerogels	PVA fibre	0.320	[46]
	PVA/CNF (1:1.5)	0.038	[30]
	PVA/HAP aerogel (3:1)	0.039	[47]
	PVA/ZIF-8 aerogel (1:1)	0.036	[48]

## Data Availability

The data presented in this study are available on request from the corresponding author.

## Supplementary Materials

The following supporting information can be downloaded at: https://www.mdpi.com/article/10.3390/nano14090745/s1, Videos S1–S3. Figure S1. (a) SEM and (b) AFM images of GO nanosheets. Figure S2. Raman mapping of PGO 2 aerogel demonstrating the distribution of GO nanosheets. Figure S3. SEM images of A-PGO 2 along (a) longitudinal, and (b) transverse directions.
