# Supplementary material for: Development of Light, Strong, and Water-Resistant PVA Composite Aerogels
Source: Nanomaterials (Basel). 2024 Apr 24;14(9):745. doi: 10.3390/nano14090745 (PMC11085475; doi:10.3390/nano14090745)
Supplement: Supplementary file 1 [file nanomaterials-14-00745-s001.zip › Supplementary Data.pdf]

(a) **(b)**

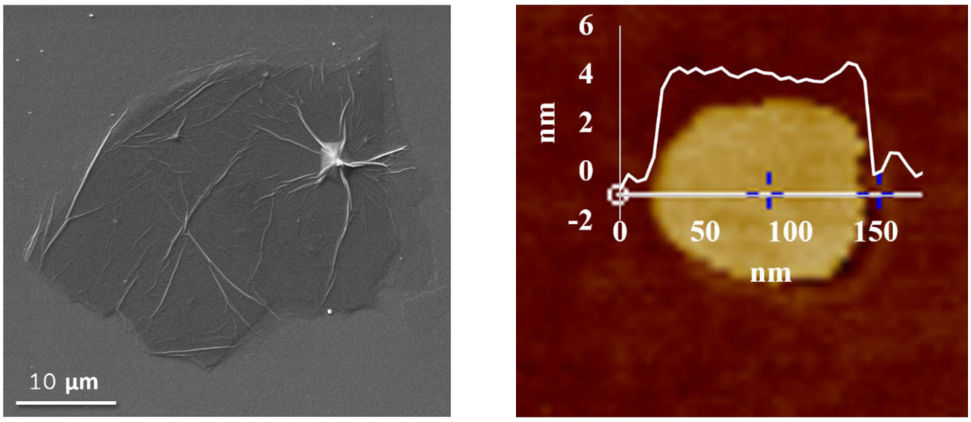

Figure S1. (a) SEM and (b) AFM images of GO nanosheets.

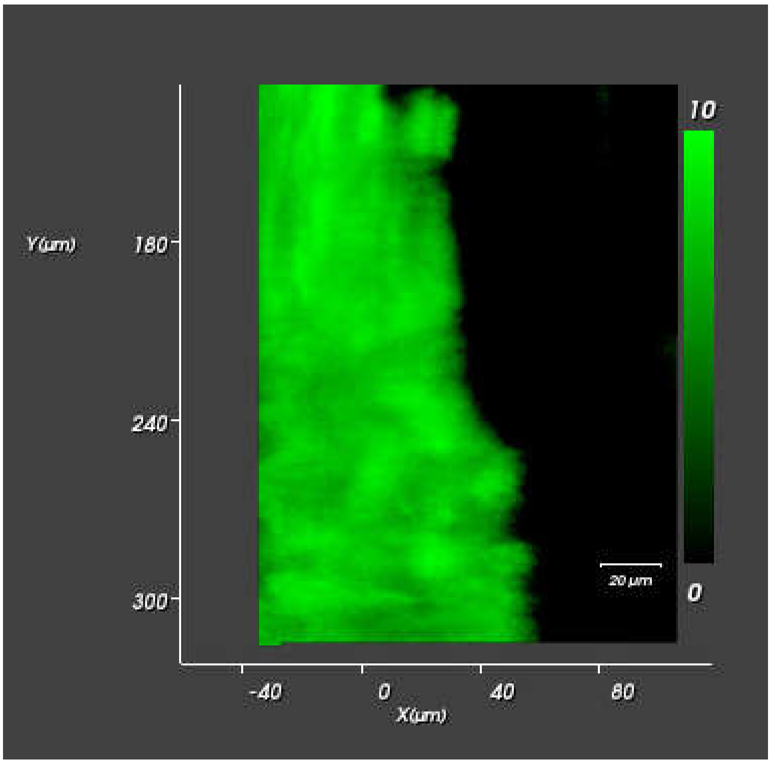

Figure S2. Raman mapping of PGO 2 aerogel demonstrating the distribution of GO nanosheets.

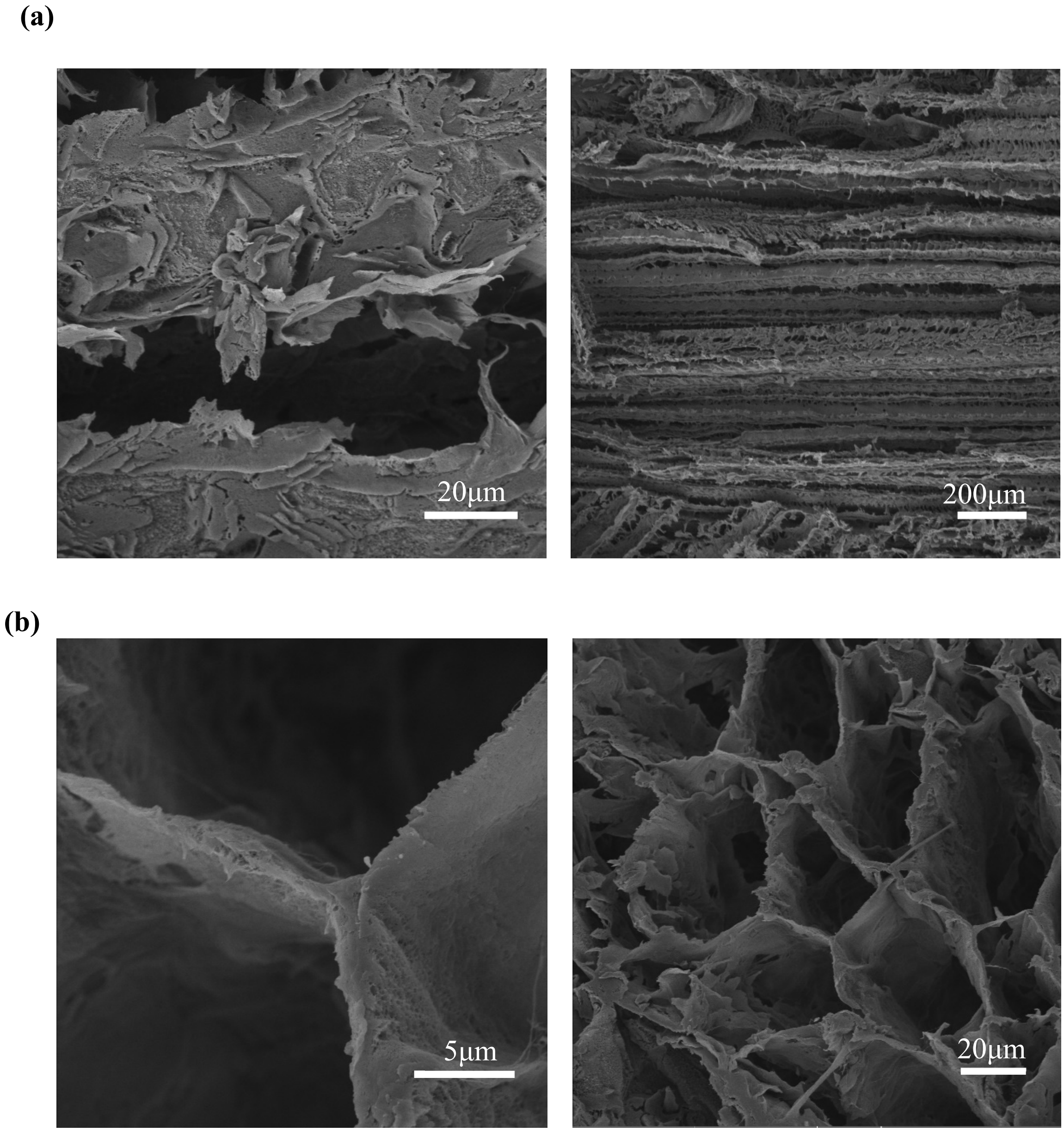

Figure S3. SEM images of A-PGO 2 along (a) longitudinal, and (b) transverse directions.
